# Pre-Survey Text Messages (SMS) Improve Participation Rate in an Australian Mobile Telephone Survey: An Experimental Study

**DOI:** 10.1371/journal.pone.0150231

**Published:** 2016-02-26

**Authors:** Eleonora Dal Grande, Catherine Ruth Chittleborough, Stefano Campostrini, Maureen Dollard, Anne Winifred Taylor

**Affiliations:** 1 Population Research and Outcome Studies, School of Medicine, The University of Adelaide, Adelaide, Australia; 2 School of Population Health, The University of Adelaide, Adelaide, Australia; 3 University Ca’ Foscari, Venice, Italy; 4 Asia Pacific Centre for Work Health and Safety, School of Psychology, Social Work & Social Policy, University of South Australia, Adelaide, Australia; University of Geneva, SWITZERLAND

## Abstract

Mobile telephone numbers are increasingly being included in household surveys samples. As approach letters cannot be sent because many do not have address details, alternatives approaches have been considered. This study assesses the effectiveness of sending a short message service (SMS) to a random sample of mobile telephone numbers to increase response rates. A simple random sample of 9000 Australian mobile telephone numbers: 4500 were randomly assigned to be sent a pre-notification SMS, and the remaining 4500 did not have a SMS sent. Adults aged 18 years and over, and currently in paid employment, were eligible to participate. American Association for Public Opinion Research formulas were used to calculated response cooperation and refusal rates. Response and cooperation rate were higher for the SMS groups (12.4% and 28.6% respectively) than the group with no SMS (7.7% and 16.0%). Refusal rates were lower for the SMS group (27.3%) than the group with no SMS (35.9%). When asked, 85.8% of the pre-notification group indicated they remembered receiving a SMS about the study. Sending a pre-notification SMS is effective in improving participation in population-based surveys. Response rates were increased by 60% and cooperation rates by 79%.

## 1 Introduction

Many chronic disease and risk factor surveillance systems in Australia use the telephone as an efficient way to collect information. The telecommunication industry has undergone many changes over the last 15 years which has had an impact on traditional landline-based surveys. Increased non-coverage and declining participation has required telephone survey researchers to adjust their methodology.[1, 2] There has been an increase in mobile-only households in Australia and internationally,[3–5] and this has had an impact on the coverage of landline-based surveys.[2, 6] As a result many systems are incorporating mobile telephone samples into their surveys resulting in dual-frame sampling methods.[2, 7–9]

Incorporating mobile telephones samples into population surveys has brought challenges in both sampling and participation.[10, 11] In Australia, there is the difficulty in obtaining a representative sampling frame of mobile telephone numbers since they are rarely listed (7.3% of mobile telephone owners in South Australia are listed).[12] Several studies in Australia used a random-digit dial (RDD) list of mobile telephone numbers[13, 14] but this is compromised as mobile telephone numbers do not include address details or geographical location. As such, sending a primary approach letter (PAL) is not possible for geographically restricted surveys. Landline telephone numbers from directory-listed sampling frames that include address details allow the option of sending a PAL, which softens the impact of unsolicited calls and has been shown to improve response rates.[15]

There are a number of factors which have influenced people’s participation in surveys using mobile telephones. The function of caller ID has contributed to this decline in response rates due to privacy concerns, survey burden and has enable the user to screen calls.[16] People are worried about the invasion of their privacy and have developed a mistrust of unsolicited calls.[17] A United States (US) study indicated that only 44% of people would let the call go to voice mail, 10% would ignore the call all together and 44% will answer the call.[18] The different ‘user culture’ associated with mobile telephones includes people regarding their mobiles as a private tool when compared to landline telephones with mobile telephones predominately used to converse with close friends and family members.[19] This makes it increasingly difficult to make ‘cold’ calls to mobile telephones.[20] Another challenge is the location at the time of data collection with landline interviews undertaken within the respondent’s home while mobile interviews can additionally be undertaken in a wide range of environments outside the home. This means interviews via mobile telephones increases cognitive burden, therefore providing additional distractions, and challenges privacy considerations which can lead to higher breakoff or refusal to participate.[21, 22] Unlike landline telephones, mobile telephones have various platforms: different operating systems, features, screen sizes, touch screens, keyboard or keypad options, different modes or formats of text messaging; all which have an impact on the way people interact or use their mobile telephones.[11] These issues are associated with lower response rates for mobile telephone interviews compared to landline telephone surveys resulting in the need for alternative methods to increase response rates.

A standard feature of mobile telephones is the ability to communicate by Short Message Service (SMS) or, more commonly known as, text messaging. In Australia, 85% of adults owning a mobile telephone indicated that they use SMS.[23] It is a relatively cheap way of communicating with a higher proportion of young people opting to SMS rather than call.[24] SMS has been used for many years in businesses as a reminder to clients of their appointment time and date.[25] This indicates the potential to incorporate SMS into survey methodology and improve response rates, especially among the difficult to reach groups such as the young and highly mobile people. Unlike PAL, SMS is considered fast, is received immediately or stored until the message is able to be read, and there is an immediate notification of a non-working number. With current available technology, SMS can be sent simultaneously to a large number of people.

Few studies have tested the effectiveness of sending pre-notification SMS to increase participation in population-based mobile telephone surveys. These previous studies indicated that there are no differences in the response rates for those who were sent a pre-notification SMS compared to those who were not.[20, 21, 26, 27] Although the response rate was not different, Steeh et al[20] found that surveys incorporating a pre-notification SMS had an increased cooperation rate (50.1% compared to 41.5%), lower refusal rate (10.3% compared to 21.1%) and fewer call attempts compared to no SMS. In a study conducted by DuBray,[26] only a third of the respondents indicated they recalled receiving a pre-notification SMS (33%) which could explain the lack of observed difference in response rate. It should be noted that these studies were conducted in the US where the receiver of the incoming SMS pays for the incoming call.[19, 20] However, the payment system in Australia and Europe is different, with cost of the SMS paid by the person or organization sending the SMS.

The current study was designed to examine the role of SMS in increasing response rates in Australia. This study was part of a broader project which included current workers.[28] Previous data collection for the project was solely based on a directory-listed landline sampling frame. Literature indicated that the proportion of currently employed adults had higher rates of mobile-only households, and limiting the sample to a directory-listed landline sampling frame would result in a lower proportion of young people, people living in lower socio-economic status (SES) areas, and renters.[3, 5] Thus, a dual-frame telephone sampling approach was considered. This involved two different telephone sampling frames: a landline telephone sample and a mobile telephone sample.

The aim of this study was to test if sending pre-notification SMS to inform users of an imminent mobile telephone call from researchers about a survey improves response rates and participation in a population-based study among mobile telephone users. Because the uptake and saturation of mobile telephones has grown so quickly since the mid2000s[3, 5], and the technology has changed and evolved over the last decade as well as people’s behaviours[29], the literature in this area is sparse and, moreover, findings from five years ago may not be relevant or applicable today.

## 2 Materials and Methods

### 2.1 Survey design and sample selection

This study is part of the Australian Workplace Barometer (AWB) project which aims to provide epidemiological evidence of Australian workplace conditions.[28] For this paper, only the methodology for the mobile telephone study will be presented. The sample frame used a randomly generated mobile telephone number supplied by Sampleworx.[12] Since the sample had no geographical marker, the sample could not be stratified by state or territory, hence, the mobile telephone sample was a random selection of mobile numbers of Australia.

Ethical approvals were obtained from the Research Ethics Committees of The University of Adelaide and the University of South Australia at each stage of the AWB project including this study to test sending a pre-notification text. Participation in the study is voluntary. Verbal informed consent was obtained from participants at the start of the interview and confirmation to continue participation in the telephone interview was obtained and recorded as yes or refusal within the questionnaire. The study was conducted via the mobile telephone and obtaining written consent or sending a primary approach letter (PAL) was not feasible due to inability and unwillingness of respondents to provide mailing address details. Upon initial contact, respondents can have a PAL mailed out if requested. Consent was recorded as a complete interview and reasons for non-participation or unable to establish contact were also recorded.

A simple random sample of 9000 mobile telephone numbers Australia-wide was selected. To determine the effectiveness of sending a pre-notification SMS, 4500 mobile numbers were randomly selected to be sent a SMS. To be eligible for participation, respondents had to be an adult aged 18 years and over, and currently in paid employment. We assumed that the person who answered the mobile telephone was the primary user. People who were self-employed were not eligible to participate. There were no replacements for non-contactable persons. Data collection for this study occurred between 29 October 2014 and 23 February 2015. All interviews were conducted in English.

### 2.2 SMS messages

SMS messages were sent using smsglobal (www.smsglobal.com), a web messaging platform (MXT), which is a management tool to send SMS online. The MXT has options to send from a dedicated number or from words limited to 11 characters. We chose to have “Uni SA AWB” since the University of South Australia is a well-known and respected institution, and the results from an internet search using these terms provides links to AWB material. The length of the message was set at the standard 160 characters (including spaces). The 160 characters was costed as one SMS message; any more would have doubled the cost. The main aim of the message was to inform the participant that they were going to receive a call, the number that was going to be used and a free-call 1800 number to call if they had any queries. The respondents did not have the option to reply by sending a SMS. Random batches of mobile numbers were selected daily and scheduled for SMS to be sent at noon each day with the telephone call made later that evening. Smsglobal software flags SMS messages that were unsuccessfully sent, indicating that the mobile telephone number was not active and could be removed from the sample. As part of the market and social research industry standards in Australia, both sample groups had the telephone number of the caller appearing on the screen, in this case, a landline telephone number. No other information, such as “UNI SA AWB” appeared when calling to the mobile telephone. Up to three SMS were sent to the participants to obtain an interview. The follow-up SMS messages were worded almost the same as the initial SMS (see Appendix 1). For both sample groups, if there was no answer, the interviewer left a voice message if possible (see Appendix 2). Up to five call-backs were made to establish contact.

### 2.3 Socio-demographics

Socio-demographic variables included in these analyses were age group, sex, country of birth (Australia, outside Australia), educational attainment (bachelor degree level or higher, below bachelor level), and working hours (full time, part time).

### 2.4 Statistical analyses

The response rate was used to determine the effectiveness of pre-notification SMS. The final dispositions of the mobile telephone numbers were classified using the American Association for Public Opinion Research (AAPOR) standard definitions.[30] A series of outcome rates[30] were calculated to evaluate the performance between the SMS and no SMS mobile telephone groups. There are different formulas for each rate to incorporate the unknown eligibilities of some mobile telephones:

- response rates (RR): The number of complete interviews divided by the number of eligible respondents in the sample.
- cooperation rates (COOP): The number of all cases interviewed divided by all eligible respondents ever contacted.
- refusal rates (REF): The number of all respondents who refused to be interviewed, or terminated an interview, divided by all potentially eligible cases.
- contact rates (CON): The proportion of all cases in which some responsible housing unit member was reached.

Univariable analyses using chi-square tests were conducted to compare each of the outcomes rates and socio-demographic characteristics between the SMS and no SMS mobile telephone groups. In addition, to examine the representativeness of the two mobile telephone groups with regard to selected socio-demographic characteristics (prevalence (%) and 95% confidence intervals), comparisons were made against the Australian Bureau of Statistics (ABS) Census [31] data of people in paid employment (excluding self-employed).

Data analysis was conducted using SPSS Version 21.0.

## 3 Results

From the original sample of 9000 mobile telephone numbers, 3809 were ineligible due to being a non-connected number (1755), non-residential number (102), fax/modem connection (23), pager service (191) and the respondent being ineligible to participate in the survey (1738) (Table 1). Ineligible respondents were mainly due to being aged under 18 years (530) and either self-employed or not employed (1208). This left an eligible sample of 5191 mobile telephone numbers: 2566 that were sent a pre-notification SMS and 2625 that were not sent a pre-notification SMS.

**Table 1:** AWB Response rates: mobile telephone sample [using American Association for Public Opinion Research standards][30].

	No pre-notification SMS	Pre-notification SMS	P value
**Interview (Category 1)**			
Complete	203	317	
**Eligible, non-interview (Category 2)**			
Refusal and breakoff (terminated)	15	15	
Refusal	928	685	
Non-contact			
Respondent never available	1	2	
Answering machine household-message left	27	9	
Other, non-refusals			
Physically or mentally unable/incompetent	10	9	
Language problem	113	84	
**Unknown eligibility, non-interview (Category 3)**			
Always busy	2	0	
No answer	1326	1445	
**Not eligible (Category 4)**			
Fax/data line	9	14	
Disconnected number	891	864	
Special technological circumstances			
Pager	95	96	
Non-residential number	60	42	
No eligible respondent	820	918	
**Total phone numbers used**	4500	4500	
I = Complete Interviews (1.1)	203	317	
P = Partial Interviews (1.2)	0	0	
R = Refusal and break off (2.1)	943	700	
NC = Non Contact (2.2)	28	11	
O = Other (2.0, 2.3)	123	93	
Calculating e:	0.41	0.37	
UH = Unknown Household (3.1)	1328	1445	
UO = Unknown other (3.2–3.9)	0	0	
**Response Rate 1 [& 2]** I/(I+P) + (R+NC+O) + (UH+UO)	7.7	12.4	<0.001
**Response Rate 3 [& 4]** I/((I+P) + (R+NC+O) + e(UH+UO))	11.0	19.2	<0.001
**Cooperation Rate 1 [& 2]** I/(I+P)+R+O)	16.0	28.6	<0.001
**Cooperation Rate 3 [& 4]** I/((I+P)+R))	17.7	31.2	<0.001
**Refusal Rate 1** R/((I+P)+(R+NC+O) + UH + UO))	35.9	27.3	<0.001
**Refusal Rate 2** R/((I+P)+(R+NC+O) + e(UH + UO))	51.2	42.4	<0.001
**Refusal Rate 3** R/((I+P)+(R+NC+O))	72.7	62.4	<0.001
**Contact Rate 1** (I+P)+R+O / (I+P)+R+O+NC+ (UH + UO)	48.3	43.3	<0.001
**Contact Rate 2** (I+P)+R+O / (I+P)+R+O+NC + e(UH+UO)	69.0	67.2	0.27
**Contact Rate 3** (I+P)+R+O / (I+P)+R+O+NC	97.8	99.0	0.02

e is the estimated proportion of cases of unknown eligibility that are eligible.[30] Enter a different value or accept the estimate in this line as a default. This estimate is based on the proportion of eligible units among all units in the sample for which a definitive determination of status was obtained (a conservative estimate).

A total of 526 eligible adults participated in the survey; 60.4% were sent a pre-notification SMS (318) and 39.5% were not (208). The response rate was 12.4% (RR1) for the mobile sample that was sent a pre-notification SMS and 7.7% for the sample that was not (Table 1). The SMS mobile telephone group had a higher cooperation rate (COOP1, 28.6% versus 16.0%) and a lower refusal rate (REF1, 27.3% versus 35.9%) compared to the mobile telephone group with no SMS.

The average time of the two surveys did not differ: 32.8 minutes (standard deviation = 7.62) for respondents who received a SMS and 33.2 minutes (standard deviation = 7.62) for those who did not.

Even though the toll-free 1800 number was given in the SMS, only seven people rang to opt-out of the survey. Statistics on the number of people using the 1800 number to query the survey were not recorded. When asked, 85.8% of the pre-notification SMS group remembered receiving a SMS about the study. There were no differences between males and females in the proportion of recall, however, recall was lower amongst respondents aged 18 to 24 years (80.5%) and 55 and years and over (81.4%).

When examined against the ABS Census population (Table 2), there were no differences in the two mobile telephone groups by sex. There was no clear pattern by age groups for either mobile telephone group, with some age groups close to the Census population. Even though the two mobile telephone sample groups did not differ to each other in terms of educational level and country of birth, both groups had a higher proportion of respondents with a bachelor degree or higher level of education and respondents born outside of Australia compared to the Census population. The SMS mobile telephone group had the same employment hours distribution as the Census whereas the no SMS group had a lower proportion of fulltime participants.

**Table 2:** Demographic profile by pre-notification SMS mobile telephone groups.

	ABS Censusemployed	No pre-notification SMS		Pre-notification SMS		
	%	n	%	n	%	P value
**Sex**						
Male	50.1	104	51.2 (44.4–58.0)	160	50.5 (45.0–55.9)	0.87
Female	49.9	99	48.8 (42.0–55.6)	157	49.5 (44.1–55.0)	
**Age groups**						
18–24 years	15.2	32	15.9 (11.5–21.6)	41	12.9 (9.7–17.1)	0.22
25–34 years	24.4	41	20.4 (15.4–26.5)	81	25.6 (21.1–30.6)	
35–44 years	23.3	47	23.4 (18.1–29.7)	57	18.0 (14.1–22.6)	
45–54 years	21.9	53	26.4 (20.8–32.9)	79	24.9 (20.5–30.0)	
55–64 years	13.1	19	9.5 (6.1–14.3)	47	14.8 (11.3–19.2)	
65+	2.1	9	4.5 (2.4–8.3)	12	3.8 (2.2–6.5)	
**Education level**						
Bachelor degree or higher	23.8	83	40.9 (34.4–47.8)	115	36.3 (31.2–41.7)	0.29
Below bachelor level	76.2	120	59.1 (52.2–65.6)	202	63.7 (58.3–68.8)	
**Country of birth**						
Australia	72.4	132	65.3 (58.6–71.6)	218	68.8 (63.5–73.6)	0.42
Outside Australia	27.6	70	34.7 (28.4–41.4)	99	31.2 (26.4–36.5)	
**Working hours**						
Full time	70.4	120	60.9 (54.0–67.5)	220	70.7 (65.5–75.5)	0.02
Part time	29.6	77	39.1 (32.5–46.0)	91	29.3 (24.5–34.5)	

ABS: Australian Bureau of Statistics [31]

## 4 Discussion

The results of our study showed that sending a pre-notification SMS was effective in improving participation in population-based surveys using a RDD list of mobile telephones as the sampling frame. Although the absolute response rate was low, this feature increased the response rates (RR1) by 60%, cooperation rate (COOP1) by 79% and lowered refusal rates (REF1) by 24%. Our study contradicts other results in the literature, with our study indicating an improvement in response rates in the SMS mobile telephone group.[20, 21, 26, 27] A possible reason for this could be that this study was conducted in Australia and there could be different legal or legislation issues, and cultural differences in familiarity and ability in using SMS features in mobile telephones. In addition, the different payment system in Australia, whereby the researcher pays for both SMS and call to the participant, could make this methodology more acceptable. In comparison, for example, in the US, the receiver of the SMS or call to the mobile telephone incurs the cost, not the sender or person making the call.[32] Therefore, the participants in our study were not refusing to participate because of cost. Three of these previous studies[20, 21, 27] were conducted over eight years ago (2004 to 2007) where the SMS features were most likely not as widely used or familiar, or a standard feature of the device. Also our participants were more likely to recall receiving the SMS (85.8%) compared to a recent study by DuBray (33%) which could explain why they found no differences in the response rates.[26] Although, Steeh et al[20] found no differences in the response rates, our findings were similar to theirs in terms of increased cooperation rates and decreased refusal rates.

This study is unique as it is the first of its kind in Australia, and its strength lies in its population approach using a large Australia-wide sample rather than a convenient sample. It is also timely and has been undertaken in a population which is more accepting of SMS in terms of usage and familiarity; in 2011, 78% of Australians who owned a mobile telephone regularly used SMS and this increased to 85% in 2014.[23] However, there are weaknesses associated with this study. Since our SMS was sent in English only and our study was limited to people who were currently employed, the majority were aged between 18 and 64 years, and therefore we cannot infer that the results are generalizable to the whole population. Up to 3% of Australians, 10 years and over, do not speak English well or not at all (2.5% do not speak English well or and 0.5% do not speak English at all (0.5%) [31], and an Australian study found that 3.7% of people aged 15 to 74 years had poor literacy skills (below Level 1) [33]. As Australia’s population is linguistically diverse, with 400 languages spoken, including Indigenous languages, it is not possible to send SMS in different languages. It is not known for Australian migrants who do not understand English how they overcome these issues, but some migrants have use free online translation softwares, such as Google Tranlate®, or dictionary apps, such as Bing Translator® to overcome the language barrier. Given this, we can assume there still will be up to 3.7% of the population with poor literacy skills that would not understand our SMS message and mostly likely not participate in general population surveys.

There are other limitations in regards to the application of using SMS for surveys. These include the additional cost in sending a SMS (0.10c per SMS) and administration, although this cost was lower than sending a PAL (paper, printing, postage and envelopes). To limit recall bias, the SMS was sent during the morning of the planned telephone call. As a result, this created additional daily workload for administration staff. Feedback from the administration staff found the process relatively easy using appropriate software. Also, to minimize cost, the length of the message was limited to 160 maximum characters; any more would have doubled the cost per SMS (0.20c per SMS with maximum of 320 characters). This means, unlike the tradition PAL, our SMS did not include more detail about who was conducting the study, justify the nature of the study, the role of the respondent, the importance of the respondent’s participation, and assurances of anonymity and confidentiality. The SMS method had an added benefit in providing the status of the mobile telephone number immediately after sending the SMS so that disconnected numbers could be removed from the sample saving costs in terms of interviewer time.

The concept of the pre-notification SMS is to eliminate the element of surprise or misunderstanding and to indicate that the call is legitimate. The SMS was also designed to overcome the problem of an unrecognized telephone number on the caller-ID that may be ignored. This is important in today’s culture of increasing mistrust of unsolicited calls and provides the respondent the option to investigate the legitimacy of the incoming number if they wish. Unlike landline telephones, mobile telephones are usually attached to a person and not a household. Our study had a very small number of people using the toll-free 1800 number to opt out which might suggest that people did not feel suspicious about our study. General feedback from the interviewers found that pre-notification SMS made a minor impact on the respondents being more receptive or interested in the survey. We did not provide the option for the respondents to reply by sending a SMS as this was seen as an easy way to opt-out and also to avoid nuisance or abusive SMSs.

It should be noted that this is a relatively new surveying area and the general population may not be familiar with receiving research market calls as they do on landlines. Furthermore, unlike other methods such as landline or online internet, mobile telephones have not yet been extensively overused by marketing companies and spammers. Continual monitoring of response rates for population surveys using a mobile telephone sample is required to see if the benefit is upheld. Using SMS is one feature of mobile telephones we can utilize. Mobile telephones are continually evolving with smartphones being the next generation that researchers can explore possibilities of incorporating other types of pre-notification messages such as links embedded to webpage with additional details of the study, voice messages in which the respondent can choose their language, and the use of multimedia message.

## 5 Conclusion

This study has shown the benefits of sending a pre-notification SMS with improvements in response and cooperation rates, and reduction in refusal rates, for population surveys using mobile telephones. Further research is needed to apply this method to incorporate the total population to determine if the results found in this study are generalizable to the whole of the population. In addition, given that mobile telephone technology is continually changing and the general population’s behaviours are also changing with it, these studies need to be conducted regularly.

## Appendix 1

SMS received from “Uni SA AWB”.

**Initial SMS:**

You have been chosen to participate in an important Australian Research Council survey. An interviewer will ring on 9999 9999. RSCHD 1800999999. Thank you.

**Follow-up SMS:**

You have been chosen to participate in an important Australian Research Council survey. An interviewer will ring on 9999 9999. RSCHD 1800999999. Thank you.

## Appendix 2

Hi, my name is ……. Calling on behalf of the University of South Australia. Sorry we missed you but we will try calling again at a later date.

## Abbreviations

AAPOR: American Association for Public Opinion Research
AWB: Australian Workplace Barometer
CON: contact rates
COOP: cooperation rates
MXT: web messaging platform
PAL: primary approach letter
RDD: random-digit dial
REF: refusal rates
RR: response rates
SMS: Short Message Service
